# Diagnosis of Partial Retrograde Ejaculation in Non-Azoospermic Infertile Men with Low Semen Volume

**DOI:** 10.1371/journal.pone.0168742

**Published:** 2017-01-06

**Authors:** Roger Mieusset, Marie Walschaerts, François Isus, Thierry Almont, Myriam Daudin, Safouane M. Hamdi

**Affiliations:** 1 Université Toulouse III—Paul Sabatier, Groupe de Recherche en Fertilité Humaine (Human Fertility Research Group), Toulouse, France; 2 Andrologie—Médecine de la Reproduction, Hôpital Paule de Viguier, CHU de Toulouse, Toulouse, France; 3 Explorations biologiques—Médecine de la Reproduction, Hôpital Paule de Viguier, CHU de Toulouse, Toulouse, France; University of Texas at Austin Dell Medical School, UNITED STATES

## Abstract

In non-azoospermic patients with low semen volume (LSV), looking for partial retrograde ejaculation (PRE) by searching sperm in the postejaculatory urine (PEU) is required. The use of a retro-ejaculatory index (R-ratio) was suggested to define PRE, but none of the studies indicated a specific threshold above which PRE must be considered. Our objective was to propose a threshold value for the R-ratio as indicative of PRE in patients with LSV selected to be devoid of any known causes or risk factors for retrograde ejaculation or LSV. Among our data base (2000–2009) including 632 patients with PEU, 245 male patients from infertile couples who had had a first semen analysis with LSV (< 2mL) and a second semen analysis associated with PEU, were selected on the previous criteria. A prospective control group was randomly constituted (2007–2008) of 162 first consulting male patients from infertile couples, with a normal semen volume (≥ 2mL) on a first semen analysis and who accepted to collect PEU with their usual second semen analysis, selected on the previous criteria. To define an R-ratio threshold indicative of PRE, we used a ROC curve analysis and a regression tree based on a classification and regression tree (CART) algorithm. Of the 245 LSV patients, 146 still presented low semen volume (< 2 mL) on the second semen analysis. From the use of the CART algorithm, two low (1.5% and 2.8%) and two high R-values (7.1% and 8.3%) were defined, according to the lower reference limit for semen volume of 2.0 mL (WHO 1999) or 1.5 mL (WHO 2010) respectively. As only one or no patient with normal semen volume was observed above the two high R-values, we suggest an R-value higher than the range of [7.1–8.3]% as indicative of PRE until confirmation by a prospective multicenter study.

## Introduction

Ejaculation is the forceful propulsion of seminal fluid out of the body that consists in the synchronized succession of physiological events with two distinct phases, emission and expulsion. Organs involved in the emission phase comprise the distal epididymides, vasa deferentia, deferential ampullas, seminal vesicles, prostate gland and bulbourethral glands. Organs and anatomical structures participating in expulsion are the internal urethral sphincter (or bladder neck), composed of smooth muscle cells; the urethra, surrounded over about half of its length by circular striated muscle forming the external urethral sphincter; and the pelviperineal striated muscles, including levator ani, ischiocavernosus and bulbospongiosus muscles [1, 2].

The first step in the emission phase is the closure, by firm contraction, of bladder neck to prevent retrograde flow of the seminal fluid backward into the bladder. This is followed by the ejection of prostatic secretions into the prostatic urethra together with the sperm from the vasa deferentia and deferential ampullas, and finally the seminal vesicle secretions. Once emission phase is completed, saccadic expulsion of semen through the urethral meatus is caused by synchronized rhythmic contractions of the pelviperineal striated muscles -with a key role for the bulbospongiosus muscle- and intense contractions interrupted by silence periods of the external urethral sphincter. To achieve antegrade semen expulsion, the bladder neck remains closed; whereas the external urethral sphincter is open [1–3].

Disorders of ejaculation can be classified along a spectrum ranging from premature ejaculation, through delayed ejaculation to complete anejaculation along with retrograde ejaculation [4]. Retrograde ejaculation corresponds to the failure of the bladder neck to close resulting in reflux of semen into the bladder. This results in a low-volume ejaculate and a low or null sperm count [5]. The most known pathology associated with retrograde ejaculation is spinal cord injury (SCI). In SCI men ejaculation is strongly impaired; only 16% of them can ejaculate through sexual stimulation, while 52% require penile vibratory stimulation. Antegrade ejaculation is most commonly found (65%). Retrograde ejaculation presented as pure retrograde ejaculation in 17–29% of SCI men; or associated with antegrade ejaculation in 16% [6, 7] corresponding to partial retrograde ejaculation (PRE), with low semen volume. A search for spermatozoa in urine after ejaculation (postejaculatory urine; PEU) to determine presence of PRE is also recommended in infertile patients presenting with low semen volume [8–10].

Search for the presence of sperm in urine collected *before* ejaculation indicates that no sperm were recovered in fecund or infertile men [11, 12]. In urine collected *after* ejaculation (PEU), at least one sperm was observed in 60 to 88% of fertile or fecund men [8, 12, 13] and in 65 to 98% of infertile men depending on the study [8, 11, 13, 14]. The most plausible explanation is that sperm remaining in the urethra after ejaculation are washed out in the PEU [11–13].

However, taken alone, the only presence of sperm in the PEU does not indicate PRE [14]. The use of a retro-ejaculatory index (R-ratio), which expresses the total number of sperm recovered in PEU as a percentage of the total number of sperm found in both semen and PEU, has been suggested to define PRE [8, 13, 14]. But none of these studies indicate a specific threshold above which PRE must be considered [15].

Based on male partners of infertile couples selected to be devoid of any known risk factors for retrograde ejaculation or low semen volume, the main objective of the present study was to define a threshold for the R-ratio as indicative of the presence of an abnormal number of sperm in PEU, i.e. PRE, in non-azoospermic patients presenting with low semen volume.

## Materials and Methods

The retrospective part of the study complies with the French Law of public health about clinical research. For the prospective part, all co-authors are aware about the need for both a review and an approval by an IRB. However, in mid-2006, when this study was planned, there was no IRB in our Universitary hospital (it was operational on 2010). Since urine and semen analyses are non-invasive and routine-care lab tests, clinicians considered that miction after masturbation was not a situation of risk for men’s health. So we applied standard ethical requirements of clinical research i.e. providing complete information about the study to the patients and obtaining their free consent. The study was presented again and a posteriori to the IRB which cannot give an opinion as it did not exist when the prospective study started (IRB, 5 May 2015, CHU Toulouse Universitary Hospital). Patients files and data were anonymized before analysis by the biostatistician (coauthor MW).

### Patients

**Fig 1:**
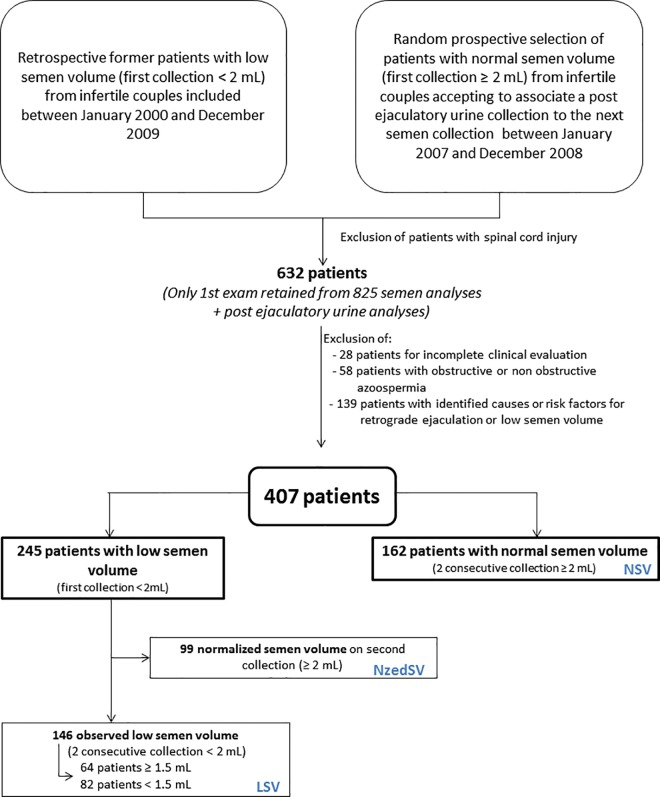
Flowchart and design of the study.

All the men included were the male partners of infertile couples referred to our centre for andrological check-up between January 2000 and December 2009. This population consisted of two groups of patients who were all included in our database (**Fig 1**):

*Prospective normal semen volume patients*: men presenting a normal semen volume (≥ 2 mL) randomly selected among the 1569 male partners of infertile couples seen in our center for the first time between January 2007 and December 2008. The whole process for this prospective part of the study was the following: after clinical examination, and without knowledge of the results of an eventual previous semen analysis, one out of two male partners of infertile couples seen in our center for the first time between January 2007 and December 2008 were randomly informed of the aims of the study and asked to provide a urine sample after ejaculation for the PEU test by one of the practitioners. They were free to accept or refuse, being informed that their choice will be without any consequences on the following care. None of the informed patient refused to collect urine after masturbation.

*Retrospective former low semen volume patients*: men who presented low semen volume (< 2 mL [16]) on a previous semen analysis (mainly carried out in another center) and who underwent a second semen collection associated with PEU in our center. All the patients were selected by a database search for semen analysis associated with a PEU test from January 2000 to December 2009. Firstly, spinal cord injury patients were excluded after identification through their medical practitioner, as well as patients with erectile dysfunction. Among the 825 remaining semen analyses associated with a PEU test, only the first analysis was selected for each patient, leaving 632 patients. We then excluded 28 patients because of incomplete records (missing history and/or ultrasound), 58 patients because of obstructive or non-obstructive azoospermia, as well as 139 patients who presented known causes or risk factors for retrograde ejaculation or low semen volume: hormonal causes (low androgen level, hyperprolactinemia), previous pelvic and retroperitoneal surgery, reduced secretion from one (absent) or both (utricle cyst) seminal vesicles, presence of a neurological deficit (multiple sclerosis), diabetes, or pharmacological treatments (antihypertensive agents, alpha-receptor blockers, antipsychotics, antidepressants) [10, 15, 17–19]. All previous inclusion criteria were used for selecting patients with retrospective low semen volume (< 2 mL) and prospective patients with normal semen volume (≥ 2 mL). Except for azoospermia, no selection was done on any sperm parameters.

Finally, the 407 selected patients were:

- Normal semen volume (NSV) patients: 162 prospective patients with semen volume ≥ 2 mL;
- 245 retrospective low semen volume (< 2 mL) patients including: 1) Normalized semen volume (NzedSV) patients: 99 patients whom semen volume was normalized (≥ 2 mL) on the second semen collection; 2) Observed low semen volume (LSV) patients: 146 patients still having a low semen volume (< 2 mL) on their second semen collection. According to the new low reference limit for semen volume [20] 64 had a semen volume ≥ 1.5 mL and 82 < 1.5 mL.

As for any patient in our centre, all the men included underwent a standardized clinical examination to look for reproductive tract abnormalities, and a standardized check-up of their surgical or medical histories to seek risk factors for infertility [21].

### Collection and analyses of semen and urine samples

After a recommended 3–5 days of sexual abstinence, patients were asked: 1) to void their bladder, 2) to collect sperm by masturbation in a special container, then 3) to collect postejaculatory urine as soon as possible by urinating again in another container free of culture medium.

Semen analysis was performed in a single laboratory (Explorations biologiques—Médecine de la Reproduction, Hôpital Paule de Viguier, CHU Toulouse, France) according to WHO guidelines [16]. Only semen volume, semen sperm count (sSC, 10^6^/mL of semen) and total semen sperm count (sTSC, semen volume multiplied by sSC, 10^6^/ejaculate) are reported in the present article.

After assessment of urine volume, 10 μL of urine were examined between slide and cover slide. If no sperm were found, the total volume of urine was centrifuged (400 g, 10 minutes). The pellet was then examined to confirm the absence of sperm in the PEU: in this case PEU was declared negative. When at least one sperm was present in the pellet or in the 10 μL sample, PEU was declared positive.

Sperm counting in PEU: using a Malassez cell (Rogo et Cie, Arcueil, France), the urine sperm count was performed as follows: when sperm were numerous, counting was done as for semen; when at least one sperm was present, the full Malassez cell (1 mm^3^) was read and the number of sperm calculated as X sperm multiplied by 1000 = urinary sperm count (uSC, 10^6^/mL of urine). The total number of sperm in the PEU or urine total sperm count (uTSC) was calculated as uTSC = urine volume multiplied by uSC (10^6^/total volume of urine).

The total sperm count in the PEU (uTSC) was expressed as the ratio R of the total sperm count in the PEU (uTSC) plus the total sperm count in the semen (sTSC) with R = [uTSC divided by (uTSC plus sTSC)] multiplied by 100 [8]. Finally, the total amount of sperm (TAS) corresponds to the sperm collected both in the semen (sTSC) and in the PEU (uTSC), i.e. TAS = sTSC plus uTSC (10^6^).

### Statistical analysis

Data of the 245 retrospective patients with former low semen volume, including 146 with still low semen volume (LSV) and 99 with normalized semen volume (NzedSV) on the second analysis, and data of the 162 prospective normal semen volume (NSV) patients were compared using the non-parametric Mann-Whitney test for quantitative data and the Chi^2^; or Fisher exact test for qualitative data. The same tests were used to compare intergroup values in complementary analyses in which the new low reference limit for semen volume (≥ 1.5 mL [20]) was included in the group of 146 men with observed low semen volume (LSV) on a second collection. All patients files were anonymized by the biostatistician (MW) before analysis.

To define an R-ratio threshold, we used a ROC curve analyses on the 146 LSV (< 2 mL) and the 162 NSV patients and then on the 82 LSV (< 1.5 mL) and the 162 NSV patients. In addition, we used a regression tree based on a classification and regression tree (CART) algorithm [22]. This method allows defining R-ratio threshold above which the total number of sperm in PEU was considered abnormal. Unlike the ROC analysis, CART analyses produce several thresholds which best separate the population into heterogeneous groups. These two methods are complementary: the ROC analysis produces the best threshold in order to obtain correct sensibility and specificity while the CART analysis refines results allowing giving range of thresholds. Statistical analysis was performed using SAS software (version 9.3, SAS Institute, Inc.) and R software (CRAN) and the significance level was defined as 5%.

## Results

### Demographic and clinical characteristics of the 162 NSV, 99 NzedSV and 146 LSV patients

Of the 245 retrospective patients with low semen volume on a former analysis, the second semen analysis performed in our centre demonstrated that 99 had normalized their semen volume (≥ 2 mL; NzedSV patients) while 146 still presented low semen volume (< 2 mL; LSV patients) (**Fig 1**). Mean (± SD) age was higher in the 146 LSV patients (36.9 ± 7.2 years) than in the 162 NSV patients (34.1 ± 5.7 years; *P* = 0.025). There were no age differences between the 99 NzedSV (34.4 ± 5.5 years) and the two other groups.

The 162 NSV, 146 LSV, and 99 NzedSV patients did not differ with regard to mean BMI (25.2 ± 3.5, 24.9 ± 3.5 and 25.3 ± 3.0 kg/m^2^ respectively), infertility duration (33.2 ± 19.6, 39.3 ± 27.8 and 33.3 ± 22.9 months respectively) and rate of primary infertility (90%, 89% and 89% respectively). Andrological histories only differed for testicular maldescent on the right side between LSV and NSV patients, with a lower frequency for LSV (9/144; 6.2%) than for NSV patients (14/158; 8.9%; p = 0.0195). No differences were found between NSV and NzedSV patients or between LSV and NzedSV.

### Biological characteristics of the 162 NSV, 99 NzedSV and 146 LVS patients

Of the 407 infertile men included, only 27 (6.6%) had no sperm in PEU (12/162 NSV and 15/245 retrospective patients, p = 0.732). As the main objective of the study was to determine a urinary sperm threshold for partial retrograde ejaculation (PRE) in infertile men with low semen volume, the 99 NzedSV patients were not included in the following analyses (except in Table 1 and Fig 3). The 146 LSV patients had a higher sSC, uSC and uTSC (*P* = 0.002, *P* < 0.001 and *P* < 0.001) but a lower sTSC (*P* = 0.002) and TAS (*P* = 0.030) than the 162 NSV patients (**Table 1**).

**Table 1:** Post ejaculatory urine and semen characteristics in the 162 patients with normal semen volume (NSV), 99 patients with normalized semen volume (NzedSV) and 146 patients with observed low semen volume (LSV) according to WHO 1999 [16].

		Mean	SD	Median	P25	P75	P90	Min	Max
**uVolume** ^a^^b^^d^	NSV	36.01	39.23	21	12.00	46.00	81.00	0.20	195
	NzedSV	47.44	42.78	35	15.00	61.80	130.00	2.20	166
	LSV	32.70	37.36	17	9	45	80	0.40	230
**uSC (10**^**6**^**/mL)** ^a^^b^^c^^d^	NSV	0.06	0.19	0.01	0.001	0.05	0.13	0	1.70
	NzedSV	0.49	2.33	0.03	0.003	0.10	0.60	0	18
	LSV	0.82	2.76	0.14	0.01	0.58	1.48	0	30
**uTSC (10**^**6**^**)** ^a^^b^^c^^d^	NSV	1.19	3.12	0.25	0.06	0.99	2.87	0	31.20
	NzedSV	10.72	44.54	0.90	0.08	3.20	11.68	0	369
	LSV	10.23	26.41	2.06	0.38	8.40	24.90	0	238
									
**Abst. Delay (days)**	NSV	4	1	4	3	4.5	5	2	8
	NzedSV	4	1	4	3	5	5	2.5	10
	LSV	4	1	4	3	5	5	1.5	8
**sVolume (mL)** ^a^^b^^c^^d^	NSV	3.70	1.34	3.30	2.80	4.40	5.60	2	8.50
	NzedSV	2.75	0.66	2.60	2.20	3.10	3.80	2.00	4.90
	LSV	1.31	0.38	1.40	1.00	1.60	1.80	0.30	1.90
**sSC (10**^**6**^**/mL)** ^a^^c^	NSV	43.23	52.68	24	8.10	59.50	110.00	0.01	382
	NzedSV	57.30	81.41	30.00	9.20	67.00	140.00	0.01	544
	LSV	74.79	96.24	39.50	15.30	98.00	191.00	0.01	648
**sTSC (10**^**6**^**)** ^a^^c^^d^	NSV	144.49	159.00	92.45	27.30	198.75	349.60	0.04	764
	NzedSV	149	191.57	74.48	24.50	243.88	388.00	0.02	1196.80
	LSV	101.29	147.38	53.99	18.70	121.20	268.60	0.01	1101.60
									
**TAS (10**^**6**^**)** ^c^	NSV	145.68	160.13	93.68	27.84	200.25	349.74	0.04	764.97
	NzedSV	160.08	202.99	89.08	26.18	249.32	391.24	0.02	1203.40
	LSV	111.52	154.31	63.61	23.94	137.06	281.82	0.09	1150.51

NSV, patients with Normal semen volume (≥ 2 mL); NzedSV, patients who normalized their semen volume (≥ 2 mL) on the second collection; LSV, patients with still low semen volume (< 2 mL) on the second collection; uVolume, urine volume; uSC, urine sperm count; uTSC, urine total sperm count (uVolume multiplied by uSC); Abst Delay, abstinence delay; sVolume, semen volume; sSC, semen sperm count; sTSC = semen total sperm count (sVolume multiplied by sSC); TAS = total amount of sperm (uTSC plus sTSC)

^a^ p < 0.05 between the groups NSV, NzedSV and LSV

^b^ p < 0.05 between groups NSV and NzedSV

^c^ p < 0.05 between groups NSV and LSV

^d^ p < 0.05 between groups NzedSV and LSV.

### Inclusion of the new lower reference limit for semen volume from WHO 2010

Since 1.5 mL was recently introduced by WHO in 2010 [20] as the lower reference limit for semen volume, we decided to include this new value in the 146 patients with still a semen volume < 2mL at their second analysis: 64 had a semen volume [1.5–2 mL [and 82 < 1.5 mL. These two subgroups differed on semen volume, but also on uTSC (higher when semen volume < 1.5 mL). Semen and PEU data were similar to those observed when using the lower reference limit for semen volume according to WHO 1999 [16] (< 2 mL) with few differences that could result from lower numbers of patients in some groups. For instance, patients a with a semen volume [1.5–2 mL [had lower semen volume (p < 0.001) and higher sSC (p = 0.039), as well as lower urine volume (p = 0.0307) and higher uSC (p < 0.001) and uTSC (p < 0.001) in PEU than NSV patients. Patients a with a semen volume < 1.5 had lower semen volume (p < 0.001) and sTSC (p = 0.001) but higher sSC (p = 0.004), as well as higher uSC (p < 0.001) and uTSC (p < 0.001) in PEU than NSV patients. All data are reported in **S1 Table.**

### Total number of sperm present in the PEU expressed as an R-ratio

As the main objective of the study was to determine a urinary sperm threshold for partial retrograde ejaculation (PRE) in infertile men with low semen volume, only the 146 SLV and the 162 NSV patients (n = 308) were used to evaluate the R-ratio (R = [uTSC divided by (uTSC plus sTSC)] multiplied by 100).

In a first step, the optimal R-value threshold was obtained from ROC plot analysis among the 162 NSV patients (≥ 2mL [16]) and the 146 LSV patients < 2mL; then, among the 162 NSV patients and the 82 LSV patients ≤ 1.5 mL [20]. Two R-values thresholds were obtained: 1.5% for semen volume < 2mL and 2.8% for semen volume < 1.5mL with a higher area under the curve higher for this second analysis (**Table 2**).

**Table 2:** Optimal R-value thresholds obtained from ROC plot for the 162 patients with normal semen volume (NSV; ≥2 mL) versus 146 patients with observed low semen volume (<2 mL) and 82 patients with observed low semen volume (<1.5 mL).

	R-value threshold (%)	AUC	Sp	Se	LR-	LR+
162 NSV *vs*. 146 LSV (sVolume **<** 2mL)	1.5	0.82	0.79	0.73	0.34	3.65
162 NSV *vs*. 82 LSV (sVolume < 1.5 mL)	2.8	0.87	0.68	0.89	0.16	2.78

AUC, area under the curve; Sp, specificity; Se, sensibility; LR-, likelihood ratio negative = sensibility/ (1 –specificity); LR+, likelihood ratio positive = (1 –sensibility)/specificity; NSV, patients with normal semen volume (≥ 2 mL); LSV, patients with still low semen volume on the second collection; sVolume, semen volume.

In a second step, a regression tree based on a classification and regression tree (CART) algorithm was used to refine the R-value thresholds obtained from ROC curves and to define thresholds above which the total number of sperm in PEU was considered abnormal.

**Fig 2:**
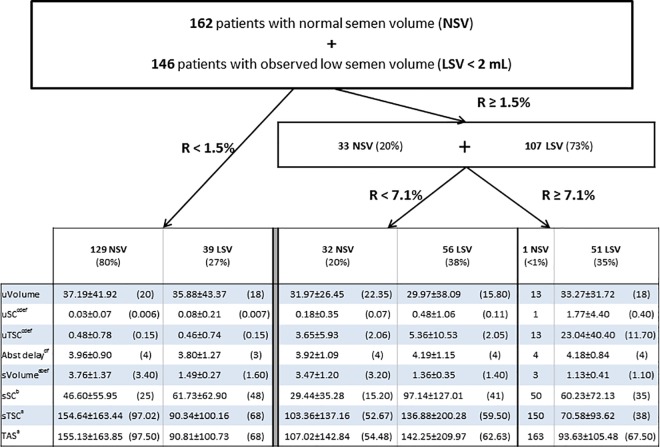
Semen and post ejaculatory urine characteristics as function of the R-value thresholds obtained from the CART procedure in 162 patients with normal semen volume (NSV) and 146 patients with observed low semen volume < 2 mL (LSV). Values are mean ± SD (median); 1.5% and 7.1%, thresholds values of R determined by the CART procedure on the 308 (162 plus 146) patients; % corresponds to number of patients/total number of patients with NSV or LSV. Three ranges of R-value classified patients: 80% of NSV patients (129/162) and 27% of LSV patients (39/146) were under an R-value of 1.5%, 20% of NSV and 38% of LSV were comprised between 1.5% and 7.1%, and less than 1% of NSV and 35% of LSV had an R-value ≥ 7.1%. R (%), [uTSC divided by (uTSC plus sTSC)] multiplied by 100 uVolume, urine volume (ml) uSC, urine sperm count (10^6^/ml) uTSC, urine total sperm count (uVolume multiplied by uSC; 10^6^) Abst Delay, abstinence delay (days) sVolume, semen volume (ml) sSC, semen sperm count (10^6^/ml) sTSC, semen total sperm count (sVolume multiplied by sSC; 10^6^) TAS, total amount of sperm (uTSC plus sTSC; 10^6^) ^a^ p < 0.05 between 129 NSV and 39 LSV ^b^ p < 0.05 between 32 NSV and 56 LSV ^c^ p < 0.05 between 129 NSV (R < 1.5%) and 32 NSV (1.5 ≤ R < 7.1%) ^d^ p < 0.05 between 39 LSV (R < 1.5%) and 56 LSV (1.5 ≤ R < 7.1%) ^e^ p < 0.05 between 56 LSV (1.5 ≤ R < 7.1%) and 51 LSV (R ≥ 7.1%) ^f^ p < 0.05 between 39 LSV (R < 1.5%) and 51 LSV (R ≥ 7.1%)

First, the CART algorithm applied to the population (n = 308) of 162 NSV patients (≥ 2 mL) and 146 LSV patients < 2mL provided two threshold values: 1.5% and 7.1% (**Fig 2**). In the 39 LSV and the 129 NSV patients under an R-value of 1.5%, PEU parameters did not differ but LSV patients had lower sTSC and TAS than NSV patients (90.3×10^6^ *versus* 154.6×10^6^, p = 0.038 and 90.8×10^6^ *versus* 155.1×10^6^, p = 0.040). For patients who had an R-value between 1.5 and 7.1%, the only differences were a lower semen volume and a higher sSC in LSV than in NSV patients (p = 0.001). With regard to PEU in LSV patients, while urine volume did not differ from one class of R-value to another, uSC and uTSC increased significantly. Semen volume of LSV patients with an R value ≥ 7.1% was lower (p = 0.006 and p < 0.001) than semen volume of LSV patients in the two other classes of R-value.

**Fig 3:**
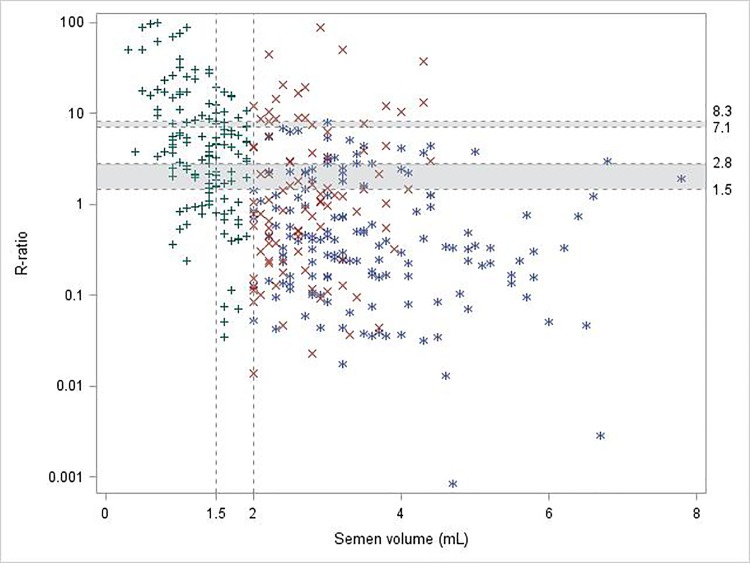
Distribution of patients according to R-values and semen volume. Reference lines were drawn on x-axis to represent WHO lower reference limits for semen volume (1.5 mL [20] and 2 mL [16]) and on y-axis to represent low and high R-value thresholds obtained from CART procedure (1.5% and 2.8%, 7.1% and 8.3% respectively). + (green), 146 patients with observed low semen volume (<2 mL) × (red), 99 patients with normalized semen volume (≥ 2 mL) * (blue), 162 patients with normal semen volume (≥ 2 mL).

Second, the CART algorithm applied to the population (n = 244) of 162 NSV patients (≥ 2 mL) and 82 LSV patients (< 1.5mL) determined two threshold values for R: 2.8% and 8.3% (**S1 Data** and **S1 Fig**).

As this analytical approaches allowed defining both a low (1.5% and 2.8%) and high (7.1% and 8.3%) R-value, the population of the 162 NVS and 146 LSV patients may be distributed according to high and low R-values and semen volume (**Fig 3**). Different subpopulations were found whether they are located above or under one of the two ranges, [7.1–8.3]% or [1.5–2.8]%.

## Discussion

In our cohort study of 407 male partners of infertile couples, 245 men were recruited with a low semen volume (< 2mL) on a former semen analysis selected on only two specific criteria never used until now: absence of any known cause or risk factors for low semen volume or retrograde ejaculation, and absence of azoospermia.

In the overall population of the 407 patients, only 27 (6.6%) had no sperm in PEU: 7.4% of NSV (≥ 2 mL) patients and 6.1% of retrospective LSV (< 2 mL) patients. This rate of 93.4% of patients with at least one sperm present in PEU is in agreement with the reported rates in infertile populations, ranging from 65% to 98.7% [8, 11, 13, 14] and in 60 to 88.7% of fertile or fecund populations [8, 12, 13]. A lower rate of sperm in the urinary sediment (5.6%) was recently reported in a large hospital-based population; however this was not PEU and information about previous ejaculation were unknown [23]. Taking together, our data confirm on a large population the previous observation that the only presence of sperm in the PEU is non informative as regard the presence of partial retrograde ejaculation (PRE) [14].

Among the 245 men with a low semen volume on a first exam, 99 (40%) did normalized their semen volume (≥ 2mL), but they differed from the 146 LSV men (still < 2 mL) with higher sTSC and lower uTSC, and from NVS men with lower semen volume and higher uTSC. No further analyses were run in this population of the 99 NzedSV patients, as it was not the objective of the present work, but they deserve further studies. From these 245 men, 60% (146) still presented a semen volume less than 2 mL on their next semen analysis; we then suggest a systematic PEU with the next semen analysis after a first one presenting LSV. The goal is to confirm LSV as a constant feature and to assess the presence of PRE. However, as sperm in the PEU of fertile or fecund men are sperm that stay in the urethral duct during the expulsion phase [11–13], this physiological process is present in both fertile and infertile men. This is why, taken alone, the only presence of sperm in the PEU does not indicate PRE [14].

To establish a diagnosis of PRE, as previously suggested [8, 13, 14] we used the retro-ejaculatory index (R-ratio) which expresses the total number of sperm recovered in PEU as a percentage of the total number of sperm found in both semen and PEU. The originality of our present study is the use of the classification and regression tree (CART) algorithm [22] in complement of the ROC curves analyses. Two low R-values (1.5% and 2.8%) and two high R-values (7.1% and 8.3%) were defined, according to the lower reference limit for semen volume of 2.0 mL [16] or 1.5 mL [20] respectively.

Regarding the two high R-values, only one or no NSV (≥ 2mL) patient was observed above. From this approach, we suggest that the two high R-values (7.1% and 8.3%) represent the upper limit of normal R-value. As the study was running before the new lower reference limit for semen volume [20], we suggest an R value higher than the range of [7.1–8.3]% as indicative of PRE. As for any threshold defined in a study, it would need to be confirmed on a prospective multicenter study. Indeed, within the 10 year-duration of semen and PEU collection in the study patients, we were able to select only 245 patients, presenting both former LSV (less than 2mL) and absence any known cause or risk factor for retrograde ejaculation or low semen volume, among about 6 000 infertile patients, i.e. an estimated prevalence of about 4%.

Finally, when distributing the population of the 162 NVS and 146 LSV patients according to high and low R-values and semen volume (**Fig 3**), different subpopulations were found whether they were located above or under one of the two ranges, [7.1–8.3]% or [1.5–2.8]%:

1) Among LSV patients, those above the range [7.1–8.3]% are considered to have true PRE. This group of patients had the lowest median semen volume (1.1 mL) probably due to the seminal liquid that remains in the posterior ureter with sperm during the expulsion phase, as recently demonstrated in some men with spinal cord injury or diabetes [24]. Such a stasis in the posterior ureter could result either from an “ejaculation dyssynergia” due to a lack of coordination between bladder neck and external sphincter [25]; or from a hormonal disturbance. Indeed, comparisons between subjects with prolactin (PRL) < 140 mU/L (139 case patients) and age-, total testosterone-, TSH-matched controls (n = 139; PRL > 140 mU/L) showed that subjects with reduced PRL had more often lower ejaculate volume, less seminal vesicle total volume either before or after ejaculation, a lower mean deferential ampullas diameter [26]. Moreover, a low PRL was associated with a weaker ability to control the ejaculatory reflex [26].

2) On the contrary, LSV patients under the low R-values [1.5–2.8]% had no PRE but lower seminal volume, lower sperm count and total sperm count than NSV patients in the same R-values. We hypothesize these LSV patients to have a factor affecting both semen volume and spermatogenesis that could be hormonal; we were not able to assess this possibility as only oligozoospermic patients have had reproductive hormones check-up. However, some patients may have exclusion of both sperm and seminal liquid secondary to a unilateral congenital absence of vas deferens–as observed in up-to 3% of azoospermic patients [27]–or acquired obstruction of one ejaculatory duct [28]. This indicates both systematic reproductive hormonal check-up and transrectal ultrasonography (TRUS) -independently of the value of sperm count- in further prospective studies to validate these hypotheses or not.

3) Two groups of patients, either LSV or NSV, were located between low and high R-values and differed only on semen volume and sperm count. We suggest these two groups of patients to be at risk of having some of their semen analyses above the high R-values, as observed in some non-permanent LSV infertile patients; a situation that could be more frequently observed with the important reduction (from 2.0 to 1.5mL) of the lower reference limit for semen volume introduced in the last WHO report [20]. A validation of this possibility must be confirmed or not by requesting at least two semen analyses associated with PEU in a prospective study involving such patients.

### Limitations

Firstly, the retrospective nature of this study is a tribute of the low frequency of permanent LSV in non-azoospermic infertile patient; a prospective multicentre study is required to confirm the present results or not. Secondly, according to the objective, the men included were not asked to take any medication or to increase their oral fluids before urine collection, or to ejaculate on a full bladder [29, 30]. Thirdly, in non-azoospermic men, LSV could result from an abnormal seminal vesicle or from ejaculatory duct abnormalities identified by TRUS except in cases of functional obstructions [31]. In the present study, TRUS was carried out only in cases of oligozoospermia and/or LSV, and patients with such abnormalities were excluded. However, as TRUS was not done in study patients without indications, i.e. men with normal semen volume and sperm count, some of them may have presented such abnormalities. Finally, as TRUS evaluation was not as precise as in recent studies [32], some potential factors of low seminal volume could have been missed such as: seminal vesicle areas of endocapsulation associated with impaired seminal vesicle emptying [33], chronic infection of the male accessory glands that may result either in lower secretory capacity of these glands [34], or in scarring and subobstruction of the ejaculatory ducts [35–37].

## Conclusion

As 60% of men without risk factors still presented a semen volume less than 2 mL on their next semen analysis, we suggest to systematically associate a PEU with the next semen analysis after a first one presenting LSV, to confirm LSV as a constant feature and to assess the presence of partial retrograde ejaculation (PRE). PRE diagnosis requires expressing sperm in postejaculatory urine (PEU) as an R-ratio as previously defined; we suggest an R-value higher than the range of [7.1–8.3]% as indicative of PRE until the results of a prospective multicenter study to be run. If there is no PRE, a TRUS is recommended to look for absence/obstruction of a seminal vesicle either isolated or associated with an absent vas deferens, whatever the sperm count.

Diagnosing PRE in men with LSV in absence of any known risk factors has potential implications in male and couple infertility. Clinical value of diagnosing PRE may allow explaining low reproductive ability in patients who do not ejaculate part of sperm leading to reducing chance of natural pregnancy. Besides, using the PEU sperm fraction in cases of LSV associated with oligozoospermia may allow moving from IVF/ICSI to intra-uterine insemination for some couples.

Knowledge value of PRE diagnosis is to decipher two subpopulations in infertile men with LSV without any risk factors for LSV or PRE. First, a specific subpopulation in which a potential dyssynergic ejaculation could exist; identifying such a population may lead further to use specific treatment to improve patients’ reproductive chances. Second, a subpopulation in which LSV without PRE is associated with impaired spermatogenesis indicating search for either a common hormonal factor or partial obstructive causes of the excurrent ducts. Taking into account the low prevalence among infertile men of LSV without any risk factors for LSV or PRE further multicentre prospective studies are required.

## Supporting Information

S1 Table Post ejaculatory urine and semen characteristics in the 146 patients with observed low semen volume (LSV) according to the WHO 2010 [20] lower reference limit for semen volume. 64 patients with semen volume in [1.5–2 [mL and 82 patients with semen volume < 1.5 mL.
(DOCX)

S1 Data Total number of sperm present in the PEU expressed as an R-ratio in the population (n = 244) of 162 NSV patients (≥ 2 mL) and 82 LSV patients (< 1.5mL). Details of statistical analyses and clinical data.
(DOCX)

S1 Fig Semen and post ejaculatory urine characteristics as function of the R-value thresholds obtained from the CART procedure. 162 patients with normal semen volume (NSV) and 82 patients with observed low semen volume < 1.5 mL (LSV). Values are mean ± SD (median). 2.8% and 8.3%, thresholds values of R determined by the CART Procedure on the 244 (162 + 82) patients. % corresponds to number of patients/total number of patients with NSV or LSV. Three ranges of R-value classified patients: 90% of NSV patients (145/162) and 32% of LSV patients (26/82) were under an R-value of 2.8%. On the contrary, an R-value higher than 8.3% was observed in none of NSV patients *versus* 41% of LSV patients (34/82).
(DOCX)
