# Supplementary material for: Diagnosis of Partial Retrograde Ejaculation in Non-Azoospermic Infertile Men with Low Semen Volume
Source: PLoS One. 2017 Jan 6;12(1):e0168742. doi: 10.1371/journal.pone.0168742 (PMC5218555; doi:10.1371/journal.pone.0168742)
Supplement: S1 Table — 64 patients with semen volume in [1.5–2 [mL and 82 patients with semen volume < 1.5 mL. (DOCX) [file pone.0168742.s001.docx]

**Supplementary data**

**S1 Table**

NSV, patients with normal semen volume (≥ 2 mL)

LSV, patients with still low semen volume (< 2 mL) on the second collection

uVolume, urine volume

uSC, urine sperm count

uTSC, urine total sperm count (uVolume multiplied by uSC)

Abst Delay, abstinence delay

sVolume, semen volume

sSC, semen sperm count

sTSC = semen total sperm count (sVolume multiplied by sSC)

TAS = total amount of sperm (uTSC plus sTSC)

^a^ p<0.05 between the groups LSV in [1.5-2[ mL and LSV < 1.5 mL;

^b^ p<0.05 between groups NSV and LSV in [1.5-2[ mL;

^c^ p<0.05 between groups NSV and LSV < 1.5 mL.
