# Supplementary material for: Diagnosis of Partial Retrograde Ejaculation in Non-Azoospermic Infertile Men with Low Semen Volume
Source: PLoS One. 2017 Jan 6;12(1):e0168742. doi: 10.1371/journal.pone.0168742 (PMC5218555; doi:10.1371/journal.pone.0168742)
Supplement: S1 Data — Details of statistical analyses and clinical data. (DOCX) [file pone.0168742.s002.docx]

**Supplementary data**

**S2 Data.**

Regarding the population (n = 244) of 162 NSV patients ≥ 2 mL and 82 LSV patients < 1.5mL (CART algorithm), LSV patients with an R-value lower than 1.5% had a shorter abstinence duration (p = 0.046 and p = 0.023) than those in the two other classes of R-value (**S2 Fig.**).

Regarding historical and clinical characteristics, differences concerned a higher frequency of infections (urinary infection or sexually transmitted diseases) in LSV < 1.5 mL (21/77) than in LSV (25/157) patients (27.3% *versus* 15.9%; p = 0.04); and a lower frequency of left clinical varicocele in LSV < 1.5 mL (16/82) than in NSV (52/159) patients (19.5% *versus* 32.7%; p = 0.03). Patients with LSV < 1.5 mL had a lower frequency of left varicocele (16/82) than patients within 1.5 and 2 mL (22/63; 19.5% *versus* 34.9%; p = 0.0244).
