# Supplementary material for: Diagnosis of Partial Retrograde Ejaculation in Non-Azoospermic Infertile Men with Low Semen Volume
Source: PLoS One. 2017 Jan 6;12(1):e0168742. doi: 10.1371/journal.pone.0168742 (PMC5218555; doi:10.1371/journal.pone.0168742)
Supplement: S1 Fig — 162 patients with normal semen volume (NSV) and 82 patients with observed low semen volume < 1.5 mL (LSV). Values are mean ± SD (median). 2.8% and 8.3%, thresholds values of R determined by the CART Procedure on the 244 (162 + 82) patients. % corresponds to number of patients/total number of patients with NSV or LSV. Three ranges of R-value classified patients: 90% of NSV patients (145/162) and 32% of LSV patients (26/82) were under an R-value of 2.8%. On the contrary, an R-value higher than 8.3% was observed in none of NSV patients versus 41% of LSV patients (34/82). (DOCX) [file pone.0168742.s003.docx]

**Supplementary data**

**S2 Fig.**


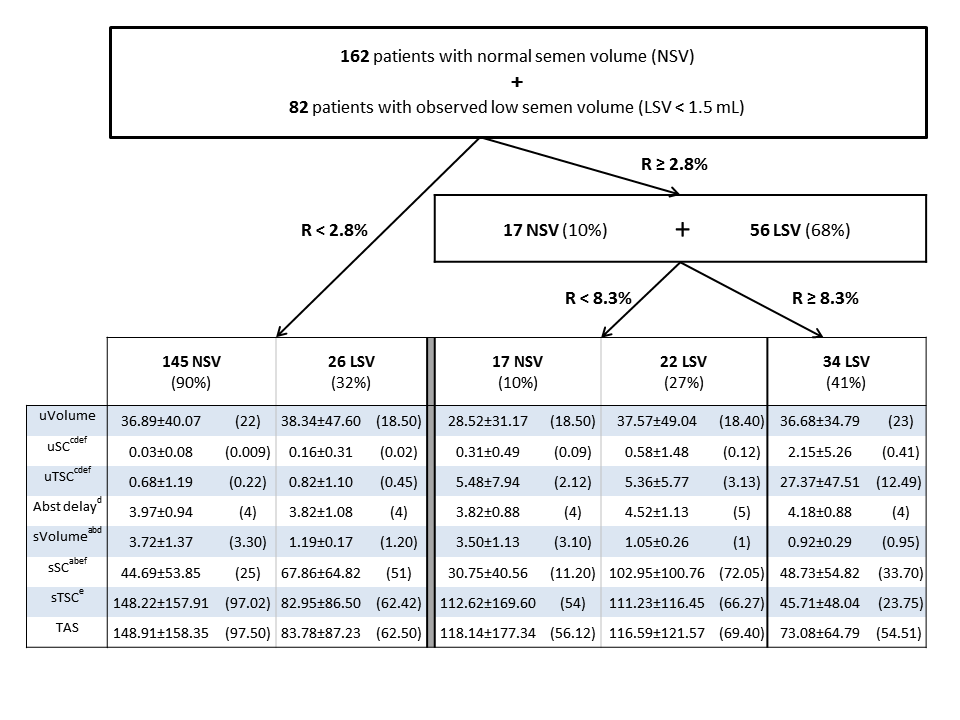


R (%), [uTSC divided by (uTSC plus sTSC)] multiplied by100

uVolume, urine volume (ml)

uSC, urine sperm count (10^6^/ml)

uTSC, urine total sperm count (uVolume multiplied by uSC; 10^6^)

Abst Delay, abstinence delay (days)

sVolume, semen volume (ml)

sSC, semen sperm count (10^6^/ml)

sTSC, semen total sperm count (sVolume multiplied by sSC; 10^6^)

TAS, total amount of sperm (uTSC plus sTSC; 10^6^)

^a^ p < 0.05 between 145 NSV and 26 LSV

^b^ p < 0.05 between 17 NSV and 22 LSV

^c^ p < 0.05 between 145 NSV (R < 2.8%) and 17 NSV (2.8 ≤ R < 8.3%)

^d^ p < 0.05 between 26 LSV (R < 2.8%) and 22 LSV (2.8 ≤ R < 8.3%)

^e^ p < 0.05 between 22 LSV (2.8≤ R < 8.3%) and 34 LSV (R ≥ 8.3%)

^f^ p < 0.05 between 26 LSV (R < 2.8%) and 34 LSV (R ≥ 8.3%)
